# Stages of Behavioral Change and Associated Factors Among Female Injecting Drug Users in Imphal, Manipur, India: An Explanatory Sequential Mixed-Methods Study

**DOI:** 10.7759/cureus.105036

**Published:** 2026-03-11

**Authors:** Lamabam H Singh, Nandu Krishna, Jayita Pal, Sanjoy Sadhukhan, Kirtesh Tiwari, Rajdeep Shaw, Aniruddha Biswas

**Affiliations:** 1 Epidemiology and Public Health, All India Institute of Hygiene and Public Health, Kolkata, IND

**Keywords:** hiv risk, northeast india, sex work and drug use, stages of change, women who inject drugs

## Abstract

Background: Female injecting drug users (FIDUs) remain a vulnerable and understudied group in Manipur’s drug-use and human immunodeficiency virus (HIV) context. Despite access to harm-reduction services, they experience gender-based stigma, unsafe injecting practices, and high health risks. Assessing readiness for change is critical for designing gender-responsive interventions.

Methods: An explanatory sequential mixed-methods design was used. Quantitative data were collected from 125 FIDUs selected using simple random sampling without replacement through a structured questionnaire incorporating the Stages of Change Readiness and Treatment Eagerness Scale-8D (SOCRATES-8D). Based on the quantitative findings, qualitative data were obtained through in-depth interviews with participants demonstrating high and low readiness for change. Quantitative data were analyzed using descriptive and inferential statistics, while qualitative data were analyzed thematically.

Results: Over half of the participants were female sex workers (53.6%), with a mean age of 29.9 ± 8.17 years. Peer pressure was the most common reason for initiating injecting drug use (48%). Low recognition of substance use as a problem was observed in 71.2%, while moderate to high ambivalence was present in 73.6%. Medium to high taking-steps scores were seen in 57.6%. In multivariable linear regression, significant predictors emerged only for the Taking Steps domain. Higher scores were associated with older age (β = 0.38, p = 0.002), being currently married (β = 0.62, p = 0.014), and earlier age at initiation of drug use (β = -0.28, p = 0.027). Daily injecting was negatively associated with taking-steps scores compared with less-than-daily injecting (β = -0.58, p = 0.019). Qualitative findings revealed normalization of drug use contributing to low recognition, persistent ambivalence related to emotional and functional dependence, and fragile action efforts undermined by withdrawal symptoms and perceived inadequate support.

Conclusion: Although motivation to change was evident, recognition remained low, and behavior change efforts were often unstable. Ambivalence, dependence, and structural barriers limited sustained change, underscoring the need for stage-matched, gender-sensitive counseling and strengthened early support within harm-reduction services.

## Introduction

Injecting drug use (IDU) remains a major public health challenge, particularly in regions located along major drug production and trafficking routes. Manipur, a northeastern state of India, shares a 358 km porous international border with Myanmar, placing it directly on the transit corridor of the “Golden Triangle,” a region historically associated with large-scale heroin production [1,2]. This geographic vulnerability has contributed to a sustained drug epidemic in the state over several decades. While the estimated national HIV prevalence in India is 0.20%, Manipur reports a substantially higher prevalence of 0.94%, with IDU continuing to be the principal driver of the HIV epidemic in the state [3]. Among people who inject drugs (PWID), HIV prevalence in Manipur remains alarmingly high, averaging around 10% statewide, with certain districts reporting prevalence as high as 19-25% in recent surveillance rounds [4].

Within this context, female injecting drug users (FIDUs) constitute a particularly vulnerable yet largely “hidden” sub-population. Evidence from Northeast India suggests that women experience a pronounced “telescoping” effect, transitioning from initial drug use to injecting in a significantly shorter period than men [5]. Initiation into IDU in this region is predominantly social in nature; over 90% of users report knowing other injectors prior to initiation, and nearly all first injections occur in the presence of peers [6]. These dynamics expose women to a compounded or “triple jeopardy” of risks, including biological vulnerability from unsafe injecting practices, heightened risk of sexual transmission of HIV, and intense socio-cultural stigma associated with both drug use and gender norms [5-7]. Unlike male IDUs, women who inject drugs are often compelled into survival sex work to sustain their dependence, and many initiate needle sharing within the first month of injecting, further amplifying their risk profile [5,6].

In response, India’s National AIDS Control Programme Phase V (NACP-V) emphasizes “differentiated prevention” strategies and gender-sensitive service delivery for key populations [8]. A notable example is the “Kapurthala Model” implemented in Punjab, which integrates harm reduction, maternal health services, and psychosocial support through a one-stop service framework [7]. This model has demonstrated that tailoring services to women’s specific needs, such as ensuring privacy, childcare support, and integrated care, significantly improves service uptake and engagement [7,8].

Despite these national-level initiatives, localized evidence from Manipur remains limited, particularly with respect to the “internal readiness” of FIDUs to initiate and sustain behavioral change. Given that drug use behaviors are deeply embedded within social networks and peer contexts, effective intervention requires an understanding not only of structural barriers but also of individual motivation and readiness for change [9]. The Transtheoretical Model of Behavior Change (TTM) offers a valuable framework in this regard. The Stages of Change Readiness and Treatment Eagerness Scale (SOCRATES) operationalizes this model across three dimensions: Recognition (acknowledgement of problematic drug use), Ambivalence (uncertainty or conflict regarding change), and Taking Steps (active engagement in behavior modification) [10,11].

Existing research in Manipur has predominantly focused on male IDUs, resulting in a substantial knowledge gap regarding the behavioral determinants and motivational profiles of women who inject drugs. Little is known about where FIDUs are positioned along the behavioral change continuum or how factors such as education, employment, caregiving responsibilities, and social support influence their capacity to move toward recovery. Current harm-reduction and targeted intervention programmes in the region are largely uniform and gender-neutral, despite the distinct social positioning, vulnerabilities, and lived realities of women who inject drugs. In the absence of context-specific evidence, programme design often assumes similar pathways and motivations for change across genders.

This study aims to assess stages of behavioral change among FIDUs in Imphal, Manipur, using the SOCRATES scale and identify associated socio-demographic and behavioral predictors, using an explanatory sequential mixed-methods design to further explore the lived experiences shaping readiness for change.

## Materials and methods

Study design and setting

This study utilized a mixed-methods explanatory sequential design, consisting of a quantitative phase followed by a qualitative phase. The research was conducted from April to December 2025 at a Drop-in Centre (DIC) located in Awang BOC, Imphal East. This centre is operated by the Nirvana Foundation, a non-governmental organization (NGO) officially supported by the National AIDS Control Organization (NACO) and the Manipur State AIDS Control Society (MSACS). The DIC provides essential harm reduction services, including the Needle Syringe Exchange Programme (NSEP) and general health support, specifically for FIDUs.

Ethical considerations

The study was conducted in accordance with the principles of the Declaration of Helsinki. Ethical approval was obtained from the All India Institute of Hygiene and Public Health Institutional Ethics Committee (IEC/2025(1)/136). Written informed consent was obtained from all participants prior to data collection after explaining the purpose of the study, procedures involved, the voluntary nature of participation, and confidentiality of the information provided.

Administrative permission to access the DIC registry and relevant serological records was obtained from the concerned NGO authority managing the centre prior to initiation of the study. The DIC registry and medical records were maintained by the NGO, and access was granted solely for research purposes.

To ensure confidentiality and anonymity, no personal identifiers were recorded in the study database. A unique study identification number was assigned to each participant. Questionnaire responses were linked to registry and serological data using this study ID only. The linkage file containing identifiers, if any, was accessible only to the principal investigator and was stored separately in password-protected form. All analyses were performed on de-identified data.

Study population and selection criteria

The study population consisted of active FIDU clients registered at the DIC. The sampling frame was established based on 326 active clients who sought services at the centre during the month of April 2025. Inclusion criteria included all willing FIDUs who were actively utilizing syringe exchange or other health support services. Participants who were unable to communicate effectively or those who were unwilling to provide informed consent were excluded from the research.

Sampling and sample size

For the quantitative strand, the sample size was calculated using Cochran’s formula for estimation of a single proportion $n_0 = \frac{Z^2 p (1 - p)}{d^2}$. A proportion (p) of 44% of individuals with drug dependence attempting to give up drug use was taken from the Magnitude of Substance Use in India (2019) report [12]. Assuming a 95% confidence level (Z = 1.96) and an absolute precision (d) of 7.5%, the initial sample size was calculated. Since the total number of active FIDUs registered at the DIC was finite (N = 326), finite population correction was applied using the formula $n = \frac{n_0}{1 + \frac{n_0 - 1}{N}}$. The sample size was further adjusted for a 10% anticipated non-response rate. The final required sample size was 125 participants. These participants were selected from the DIC active registry using simple random sampling without replacement. Selected individuals were contacted and invited to the centre specifically for the purpose of the interview and data collection.

**Figure 1:**
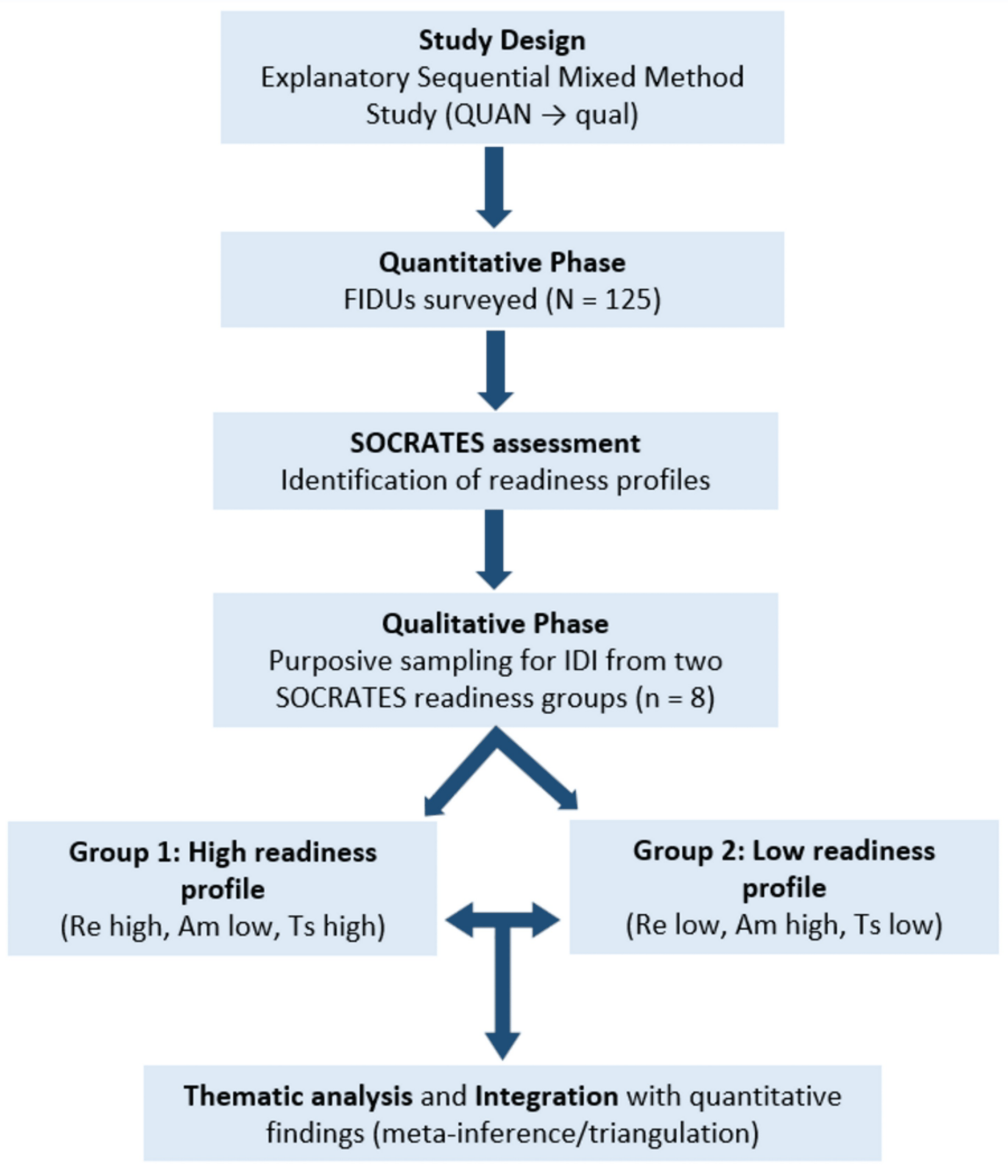
Study Design Diagram of the Explanatory Sequential Mixed-Methods Study QUAN: quantitative; FIDUs: female injecting drug users; SOCRATES: Stages of Change Readiness and Treatment Eagerness Scale; IDI: in-depth interview; Re: Recognition; Am: Ambivalence; Ts: Taking Steps

For the qualitative strand, purposive sampling with two stratified groups was employed. Participants were selected based on their quantitative results from the SOCRATES. The first group consisted of participants with high recognition and high taking steps combined with low ambivalence, representing those in the action or maintenance stages. The second group included participants with low recognition and low taking steps but high ambivalence, representing those in the pre-contemplation or contemplation stages (Figure 1).

Study tool

A pre-designed structured questionnaire was used for data collection (Appendix A). The tool comprised three components: socio-demographic characteristics, drug use history, and readiness for change, which was assessed using the SOCRATES, Version 8D. SOCRATES is a standardized 19-item instrument that measures motivation for change across three domains: Recognition, Ambivalence, and Taking Steps. Each item is rated on a five-point Likert scale ranging from “strongly disagree” to “strongly agree”, with higher scores indicating greater readiness for change.

Scoring was performed according to the standard SOCRATES manual. Item responses were summed to obtain domain-wise scores for Recognition (seven items; range 7-35), Ambivalence (four items; range 4-20), and Taking Steps (eight items; range 8-40). Higher scores reflected greater problem recognition, higher ambivalence, and greater engagement in change-related behaviors, respectively [10]. The instrument was translated into the local language using a standard forward-backward translation procedure. The translated version was reviewed by subject experts to ensure conceptual equivalence and face validity prior to administration.

Internal consistency of the translated scale was assessed in the study population using Cronbach’s alpha. The Recognition domain demonstrated high reliability (α = 0.89), and the Taking Steps domain showed excellent reliability (α = 0.94). The Ambivalence domain exhibited modest internal consistency (α = 0.63), which is considered acceptable for a brief subscale with a small number of items and reflects the heterogeneous nature of ambivalence as a construct [13].

For the qualitative component, an in-depth interview (IDI) guide was developed to explore participants’ perceptions of substance use, readiness for change, and perceived barriers and facilitators to behavior change. The guide was prepared in English, translated into the local language, and reviewed by subject experts for content validity (Appendices B-C).

Data collection

Data were collected from FIDUs registered at the Nirvana Foundation’s DIC at Awang BOC, Imphal East, using simple random sampling from the centre’s client registry. Selected participants were contacted telephonically and invited to attend the DIC on pre-specified dates for participation in the study. Prior to enrolment, all participants were informed about the objectives of the study, and written informed consent was obtained. Confidentiality was assured throughout the process.

To minimize social desirability bias, participants were explicitly informed that the investigating team was independent of service provision at the centre and that their decision to participate, or the nature of their responses, would not affect their eligibility for or access to routine services.

Quantitative interviews were conducted face-to-face by the principal investigator, a resident in public health/epidemiology, trained in research methodology and interview techniques. Interviews were carried out in a private setting within the DIC to ensure privacy and encourage candid responses. All interviews were conducted in the local language (Meiteilon-Manipuri official language) to facilitate clear communication. In addition to interview data, participants’ medical records at the facility were reviewed to ascertain their serological status for HIV, hepatitis B, and hepatitis C.

For the qualitative component, IDIs were also conducted by the primary investigator, who has formal training in qualitative research methods. Participants were selected purposively based on their readiness-for-change profiles derived from SOCRATES scores. A total of eight interviews were conducted. Interviews and analysis were conducted concurrently, and data collection continued until data saturation was achieved, with no new codes or concepts emerging.

All IDIs were conducted in a private room within the DIC in Meiteilon. With prior consent, interviews were audio-recorded to ensure accurate capture of participants’ narratives. Field notes were maintained during and immediately after each interview to document contextual details and non-verbal cues, such as facial expressions and body language, to enrich interpretation during analysis.

Reflexivity

The primary investigator conducted all interviews and maintained reflexive field notes to acknowledge their positionality and potential influence on participant responses. Regular discussions with co-investigators were held to minimize bias during data collection and interpretation.

Data analysis and integration

Quantitative data were analyzed using JAMOVI software version 2.6.44 (Jamovi Project, Sydney, Australia). Descriptive statistics were used to summarize the socio-demographic characteristics, drug use patterns, and morbidity profiles. Inferential statistical analyses were performed to examine the associations between selected independent variables and the three outcome dimensions of the SOCRATES scale (Recognition, Ambivalence, and Taking Steps); each treated as a continuous dependent variable and analyzed using linear regression.

Prior to model estimation, the assumptions of linear regression were evaluated. Predictor variables were selected a priori based on socio-demographic and behavioral factors identified from previous literature as relevant to substance use and behavioral change. The number of predictors included in the regression model was kept within acceptable limits relative to the sample size, following commonly recommended rules of at least 10 observations per predictor variable to minimize model overfitting. Normality of residuals was assessed using Q-Q plots, and homoscedasticity was examined through inspection of standardized residuals against predicted values. Multicollinearity among independent variables was assessed using variance inflation factors, with values below three indicating the absence of problematic collinearity.

Model adequacy was assessed using standard diagnostic statistics and residual plots. Adjusted regression coefficients (β) with 95% confidence intervals were reported to quantify the independent effects of predictors on each SOCRATES domain. A two-sided p-value of <0.05 was considered statistically significant.

For the qualitative data, audio-recorded interviews were transcribed verbatim in Meiteilon and subsequently translated into English. The transcripts were reviewed multiple times and cross-checked with the recordings to ensure accuracy. A manual inductive thematic analysis was conducted following a structured multi-step approach. The process involved familiarization with the data through repeated reading of transcripts, systematic generation of initial codes, organization of related codes into potential subthemes, and iterative refinement to develop overarching themes. This data-driven approach allowed patterns and meanings to emerge directly from the raw data without the use of a pre-defined coding framework. Initial coding was performed independently by two researchers to enhance credibility, and discrepancies were resolved through discussion and consensus. Themes were reviewed and refined through expert discussions to ensure that they accurately reflected participants’ lived experiences and the overall dataset.

Integration of quantitative and qualitative data

In accordance with the explanatory sequential mixed-methods design, qualitative findings were used to explain and contextualize the initial quantitative results. Integration occurred at the interpretation stage, where participant narratives elaborated on statistical patterns, enabling the development of meta-inferences that provided a comprehensive understanding of factors influencing readiness for behavioral change among FIDUs in Manipur.

Data management and quality assurance

Quantitative data were double-entered into JAMOVI and cross-checked for accuracy against original questionnaires. Qualitative interviews were transcribed verbatim, translated, and independently reviewed by two investigators to ensure fidelity. Field notes captured non-verbal cues and context. All electronic files were password-protected, and hard copies were securely stored to maintain confidentiality.

## Results

Quantitative findings

A total of 125 FIDUs participated in the study. The median age was 28 years (IQR: 24-33 years). Nearly half initiated drug use due to peer pressure (48.0%), followed by introduction through a drug-using partner (30.4%). Psychological distress accounted for 11.2% of initiations. Most participants were exclusive IDUs (86.4%), and 77.6% reported daily injecting. Monthly drug expenditure was ₹6,001-20,000 for over half of the participants (54.4%), while 13.6% spent more than ₹20,000 per month. All participants reported brown sugar/heroin (locally known as “No. 4”) as their primary injected drug (100%) (Tables 1-2).

**Table 1:** Sociodemographic Characteristics of Study Participants (N = 125) * Other ethnic groups include Maring, Chiru, Kabui, and Nepali.

Variable	Category	n	%
Age group (years)	18-24	36	28.8
	25-29	41	32.8
	30-39	33	26.4
	≥40	15	12.0
Marital status	Never married	47	37.6
	Married	17	13.6
	Divorced/separated	48	38.4
	Widowed	13	10.4
Religion	Hindu	68	54.4
	Christian	42	33.6
	Muslim	15	12.0
Ethnicity	Meitei	60	48.0
	Meitei Pangal	15	12.0
	Tangkhul	27	21.6
	Anal	10	8.0
	Other*	13	10.4
Education level	Illiterate	4	3.2
	No formal education	18	14.4
	Primary	18	14.4
	Middle	32	25.6
	Secondary	30	24.0
	Higher secondary	15	12.0
	Graduate and above	8	6.4
Occupation	Female sex worker	67	53.6
	Semi-skilled	27	21.6
	Skilled	6	4.8
	Unskilled	2	1.6
	Homemaker	7	5.6
	Unemployed	9	7.2
	Unregulated informal work	7	5.6
Living arrangement	Joint/nuclear family	67	53.6
	Rent (alone)	18	14.4
	Rent (with friends)	7	5.6
	Workplace/NGO shelter	30	24.0
	With partner	3	2.4
Monthly income (INR)	No income	17	13.6
	≤10,000	7	5.6
	10,001-20,000	58	46.4
	20,001-30,000	34	27.2
	>30,000	9	7.2

**Table 2:** Drug Use and Injection Characteristics of Study Participants (N = 125) * Psychological reasons include depression, emotional distress, and coping with separation. ** Other reasons include family conflict, easy access as a peddler, and being tricked.

Variable	Category	n	%
Age at first drug use (years)	≤17	13	10.4
	18-24	83	66.4
	≥25	29	23.2
Reason for initiation	Peer pressure	60	48.0
	Introduced by a drug-using partner	38	30.4
	Psychological reasons*	14	11.2
	Curiosity/fun	8	6.4
	Other reasons**	5	4.0
Pattern of drug use by route	Exclusive injection drug use	108	86.4
	Injection + non-injection drug use	17	13.6
Injection pattern	Daily injecting	97	77.6
	Weekly injecting	28	22.4
Monthly drug expenditure (INR)	≤6,000	40	32.0
	6,001-20,000	68	54.4
	>20,000	17	13.6

Injection-related complications were reported by 28.0% of participants, most commonly blocked veins (14.4%) and skin infections (12.8%). Acute illnesses affected 74.4%, with body ache (39.2%) and fever/headache (32.0%) being predominant. Chronic morbidities were present in 26.4%, most frequently mental health problems (15.2%). Reproductive tract symptoms were reported by 53.6% of participants. Serological testing revealed HIV positivity in 8.8%, hepatitis C reactivity in 9.6%, and hepatitis B reactivity in 7.2% (Table 3).

**Table 3:** Morbidity and Infection Profile of Study Participants (N = 125) * Multiple responses allowed.

Variable	Category	n	%
Injection-related complications (n = 35)*	Blocked vein	18	14.4
	Skin infection	16	12.8
	Overdose episode	2	1.6
Acute illness (n = 93)*	Body ache	49	39.2
	Fever/headache	40	32.0
	Cold/flu	11	8.8
	Diarrhea	5	4.0
	Respiratory illness	4	3.2
	Abdominal discomfort	7	5.6
Chronic morbidity (n = 33)*	Mental health problems	19	15.2
	Hypertension	5	4.0
	Neurological disorders	3	2.4
	Respiratory disease	3	2.4
	Chronic liver disease	2	1.6
	Thyroid disorder	1	0.8
Reproductive tract symptoms (n = 67)*	Burning sensation	35	28.0
	Foul-smelling discharge	12	9.6
	Perineal itching	11	8.8
	Lower abdominal pain	9	7.2
Hepatitis C status	Non-reactive	104	83.2
	Reactive	12	9.6
	Unknown	9	7.2
Hepatitis B status	Non-reactive	116	92.8
	Reactive	9	7.2
HIV status	Non-reactive	114	91.2
	Reactive	11	8.8

**Figure 2:**
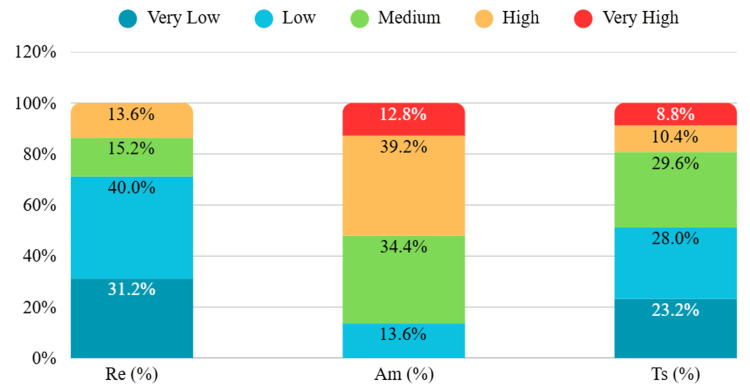
Distribution of SOCRATES Subscale Categories Among Participants (N = 125) SOCRATES: Stages of Change Readiness and Treatment Eagerness Scale; Re: Recognition; Am: Ambivalence; Ts: Taking Steps

Motivation for behaviour change, assessed using the SOCRATES scale, showed marked heterogeneity. In the Recognition domain, 71.2% of participants fell into the low or very low categories, indicating limited acknowledgment of substance-related problems. Ambivalence scores were predominantly medium or high (73.6%), reflecting substantial internal conflict regarding change. In the Taking Steps domain, 51.2% were classified as low or very low, suggesting limited active engagement in behaviour change (Figure 2).

The mean (SD) scores were 28.4 (4.42) for Recognition, 15.6 (2.38) for Ambivalence, and 29.5 (5.68) for Taking Steps. Based on standard interpretive ranges, Recognition was categorized as low, while Ambivalence and Taking Steps were in the medium range (Table 4).

**Table 4:** Score Distribution for SOCRATES Motivation-to-Change Subscales SOCRATES: Stages of Change Readiness and Treatment Eagerness Scale

Subscale	Mean (SD)	Median	Minimum	Maximum	Interpretation
Recognition (7-35)	28.4 (4.42)	28	16	35	Low
Ambivalence (4-20)	15.6 (2.38)	16	10	20	Medium
Taking Steps (8-40)	29.5 (5.68)	30	16	40	Medium

In multivariable linear regression analysis, age was positively associated with the Taking Steps domain (β = 0.38 per year; p = 0.002), and currently married participants had significantly higher Taking Steps scores compared to those not currently married (β = 0.62; p = 0.014). Higher age at first drug use was inversely associated with Taking Steps (β = -0.28; p = 0.027), while daily injecting was negatively associated compared to weekly injecting (β = -0.58; p = 0.019). No variables showed statistically significant associations with the Recognition or Ambivalence domains. The Taking Steps model demonstrated good overall fit (R^2^ = 0.275; F = 5.50, p < 0.001), explaining approximately 28% of the variance. The Recognition model showed modest but statistically significant overall fit (R^2^ = 0.140; F = 2.37, p = 0.021), although no individual predictors were statistically significant. The Ambivalence model did not demonstrate significant overall fit (R^2^ = 0.102; F = 1.65, p = 0.118). Variance inflation factors for all predictors were below 3 across models, indicating the absence of problematic multicollinearity (Table 5).

**Table 5:** Multivariable Linear Regression Analysis of SOCRATES Domains Among Female Injecting Drug Users (N = 125) SOCRATES: Stages of Change Readiness and Treatment Eagerness Scale

Variable	Recognition (Re)			Ambivalence (Am)			Taking Steps (Ts)		
	β	(95% CI)	p-value	β	(95% CI)	p-value	β	(95% CI)	p-value
Age (years)									
Per year increase	0.16	(-0.10, 0.42)	0.232	0.12	(-0.15, 0.38)	0.390	0.38	(0.15, 0.62)	0.002
Marital status									
Not currently married	1.00		1.00	1.00		1.00	1.00		1.00
Currently married	0.43	(-0.11, 0.96)	0.119	0.44	(-0.11, 0.98)	0.119	0.62	(0.13, 1.12)	0.014
Age at first drug use									
Per year increase	−0.09	(-0.36, 0.18)	0.503	-0.12	(-0.39, 0.16)	0.400	-0.28	(-0.52, -0.03)	0.027
Monthly drug expenditure (INR)									
Per unit increase	0.07	(-0.19, 0.33)	0.583	-0.02	(-0.28, 0.25)	0.909	-0.16	(-0.39, 0.08)	0.191
Reason for initiation									
Due to peer pressure	1.00		1.00	1.00		1.00	1.00		1.00
No peer pressure	0.31	(-0.06, 0.68)	0.097	0.13	(-0.25, 0.51)	0.498	0.01	(-0.33, 0.35)	0.957
Injection pattern									
Weekly injectors	1.00		1.00	1.00		1.00	1.00		1.00
Daily injector	0.47	(-0.07, 1.00)	0.085	0.41	(-0.13, 0.95)	0.137	-0.58	(-1.07, -0.10)	0.019
Reproductive tract symptoms (RTS)									
No	1.00		1.00	1.00		1.00	1.00		1.00
Yes	0.22	(-0.16, 0.60)	0.252	0.21	(-0.18, 0.60)	0.282	0.08	(-0.27, 0.43)	0.652
Any morbidity									
No	1.00		1.00	1.00		1.00	1.00		1.00
Yes	0.03	(-0.41, 0.47)	0.888	0.32	(-0.13, 0.77)	0.161	0.07	(-0.34, 0.47)	0.738

Qualitative findings

Five interrelated themes captured how FIDUs perceived harm, negotiated dependence, and engaged with behavioural change. Across themes, two experiential patterns were evident: a high-readiness group characterized by insight and sustained effort, and a low-readiness group marked by normalization of use, emotional and functional dependence, and fragmented recovery attempts.

Theme 1: Recognition Versus Rationalization of Harm (Codes: Acknowledgment of Harm; Minimization of Severity; Situational Justification)

Participants varied in how they perceived and internalized the consequences of substance use. Women in the high-readiness group demonstrated clear insight into physical, emotional, and familial harm, often linking drug use to illness and family suffering:

“I know the extent of harm… I’m HIV positive and I know how I got it.” “My mother would literally cry and beg me to stop doing drugs…...and later on I realized I was giving my family nothing but pain.”

In contrast, women in the low-readiness group acknowledged harm but reframed it as manageable or situational. Drug use was normalized as being “under control” or justified by loneliness and stress:

“As long as I’m in control, it’s okay.” “Mostly I live alone.… and I spent most of the time alone…nothing to do….so I take it.”

This theme shows that awareness of harm can coexist with continued use. Through minimization and situational justification, harm is cognitively neutralized, reducing the perceived urgency for change and contributing to low recognition.

Theme 2: Conflict Between Desire and Dependence (Codes: Fear of Life Without Drugs; Emotional Reliance; Loss of Motivation Without Use)

Many participants expressed a desire to quit while simultaneously fearing life without substances. Women described feeling emotionally empty, dull, or unable to cope during abstinence:

“I want to stop, and sometimes I really try not to take it, but when I do, I feel completely dull and empty.” “Without it, I do not feel normal anymore…. I feel like something inside me is missing.”

In the high-readiness group, this conflict was resolved through emotional clarity and acceptance of addiction:

“I did not realize when I became addicted. When I finally understood that I was an addict, it overwhelmed me and I cried."

In contrast, women in the low-readiness group remained caught between intention and dependence. Drugs functioned as emotional regulators, creating a persistent internal struggle between wanting to quit and feeling unable to cope without use.

This theme illustrates how emotional dependence sustains ambivalence. Even when harm is recognized, fear of psychological instability and loss of motivation without drugs delays commitment to change.

Theme 3: Functional Dependency in Daily Life (Codes: Use to Perform Daily Tasks; Use for Caregiving Roles; Inability to Function When Abstinent)

For many women, substance use was closely tied to their ability to function in everyday life. Drugs were described as necessary to get out of bed, complete household work, and care for children:

“If I don’t take it, I won’t be able to do household chores at all. I need it just to have enough energy to work and take care of my baby.”

Women in the high-readiness group recognized this dependence as problematic and attempted to break away from it. In contrast, those in the low-readiness group perceived drug use as essential for maintaining routine and fulfilling responsibilities.

This theme highlights that substance use was not only emotionally driven but also pragmatically embedded in daily survival. The fear of being unable to function without drugs created a powerful barrier to quitting, even when the desire to change was present.

Theme 4: Commitment and Continuity in the Recovery Journey (Codes: Sustained Engagement; Fragmented Attempts; Relapse)

Participants differed in how consistently they engaged with recovery efforts. Women in the high-readiness group described sustained commitment, often enduring severe withdrawal and remaining in treatment for prolonged periods:

“I went for detoxification and stayed there for five months.” “I have reduced my injections and started OST.”

In contrast, women in the low-readiness group reported repeated but fragmented attempts to quit, followed by relapse. Withdrawal was experienced as unbearable, and treatment settings were perceived as unsupportive:

“I have been to rehabilitation many times, but I could not bear the withdrawal. The medications they give do not work for me, and during that phase I need much more care than I receive. They simply do not take care of you properly there.”

This theme illustrates that willingness alone does not ensure recovery. Continuity is shaped by the tolerability of withdrawal and the quality of care. When treatment is experienced as punitive or inadequate, attempts remain fragile and short-lived, undermining sustained behavioural change.

Theme 5: Influence of Family and Social Environment (Codes: Family As Motivation; Family/Partner As Users; Conflicted Home Environment)

Family and social relationships played a dual role in shaping recovery. For some women, children and family members became powerful motivators for change, instilling a sense of responsibility and hope:

“I don’t want my daughter to follow this path.” “When I make efforts, they are very happy, and seeing them happy motivates me.”

For others, the home environment itself sustained substance use. Living with partners or family members who continued to use drugs normalized consumption and undermined attempts to quit:

“My husband is a chronic user… sometimes I accompany him.”

This theme highlights how recovery is embedded within intimate social contexts. Supportive relationships can strengthen resolve, while drug-using environments make abstinence socially and emotionally costly, rendering behaviour change fragile.

The joint display demonstrates how qualitative themes provide explanatory depth to the SOCRATES profiles. Low recognition is clarified by normalization and rationalization of harm; high ambivalence is explained by emotional and functional dependence on substances; and low “taking steps” is accounted for by fragile recovery attempts shaped by withdrawal suffering and perceived inadequacies in care. Thus, readiness for change among FIDUs is not merely attitudinal but is produced through an interaction of cognitive framing, emotional reliance, functional necessity, and systemic barriers (Table 6).

**Table 6:** Explanatory Integration of Quantitative and Qualitative Findings in the Explanatory Sequential Mixed-Methods Design

SOCRATES Domain	Quantitative Result (Phase I)	Qualitative Explanation (Phase II)	Explanatory Meta-Inference
Recognition	71.2% had low/very low recognition of substance use problems	Women normalized and rationalized use; harm was acknowledged but reframed as manageable or situational	The qualitative findings explain that low recognition is not due to ignorance, but to cognitive normalization, where awareness of harm coexists with justifications that reduce perceived urgency for change.
Ambivalence	73.6% had medium/high ambivalence	Drugs functioned as emotional regulators and enablers of daily life; fear of life without substances was prominent	Qualitative narratives explain high ambivalence as arising from dependence on drugs for emotional stability and routine functioning, sustaining conflict between desire to quit and perceived necessity of use.
Taking Steps	51.2% had low/very low “taking steps”	Attempts were fragile; withdrawal suffering and perceived inadequate care led to relapse and disengagement	The qualitative phase explains low action as a consequence of repeated treatment failures, severe withdrawal experiences, and low confidence in services, converting intention into cyclical, unstained efforts.

## Discussion

Readiness for change amid a high burden of harm

This mixed-methods study examined readiness for change among FIDUs in Manipur and revealed a striking disjunction between the severity of health harms and engagement with behavioural change. Despite a measurable prevalence of HIV (8.8%), hepatitis C (9.6%), and hepatitis B (7.2%), the majority of participants remained in a motivational state characterized by low Recognition, high Ambivalence, and limited active engagement in change (low “Taking Steps”) as measured by the SOCRATES tool. These findings indicate that exposure to objective harm alone is insufficient to generate behavioural change among FIDUs. Rather, readiness for change appears to be a dynamic condition shaped by cognitive framing, emotional reliance on substances, and significant systemic barriers.

The socio-demographic profile of this cohort provides an essential backdrop for understanding the motivational states of FIDUs in Manipur. Participants were relatively young, with a median age of 28 years and nearly two-thirds below the age of 30, mirroring regional data indicating that drug use in the state is increasingly concentrated in early adulthood [1]. Educational attainment was modest, with only 18.4% completing higher secondary education, suggesting that IDU often becomes established during a life stage marked by limited social and economic capital [5]. Such educational disadvantage may shape both the initial vulnerability to drug use and the subsequent capacity for sustained behavioral change.

These findings are best understood within the framework of the TTM. The predominance of low "Recognition" and high "Ambivalence" scores suggests that most participants occupy the early motivational stages of pre-contemplation and contemplation, while low "Taking Steps" scores indicate limited progression into action-oriented stages. This trajectory is deeply influenced by the relational context of initiation. More than three-quarters of participants initiated drug use before age 25, predominantly driven by peers and drug-using partners (30.4%), confirming that women’s drug use in Northeast India is embedded within intimate social networks [14].

The qualitative phase of this study elucidates the mechanisms underlying these SOCRATES profiles. Low Recognition (71.2%) was not due to a lack of awareness, but rather a cognitive normalization where substance use is reframed as a manageable coping strategy rather than a clinical pathology within the local socio-cultural context [14,15]. This normalized state, where risk is acknowledged but minimized, creates a significant barrier to the "Action" stage of the TTM. Furthermore, high Ambivalence (73.6%) was sustained by the role of drugs as emotional regulators for managing trauma and socio-political stressors. This aligns with the Self-Medication Hypothesis, where substances serve a functional psychological role, making the fear of life without the "stabilizer" a deterrent to quitting [16].

The progression from initiation to entrenched dependence, marked by 77.6% of the cohort injecting daily, often exhibits an accelerated "telescoping" effect common in women [17]. This rapid entrenchment, combined with the lack of gender-sensitive services addressing childcare and privacy, contributes to the low engagement in "Taking Steps" (51.2%). The current treatment landscape in Manipur remains predominantly male-centric, acting as a systemic deterrent for women [15,18]. These barriers reflect a state of constrained agency rather than personal indifference [18].

Structural vulnerabilities further compound this health burden. Relational disruption was a defining feature, with only 13.6% currently married and over one-third divorced or separated. Similar patterns of unstable relationships and partner loss often precede drug entry among FIDUs in this region [1,19]. Such instability, combined with limited education, shapes the limited capacity for recovery [5,20]. Furthermore, over half of the participants (53.6%) were engaged in sex work, placing them at a dual-risk intersection. International evidence suggests that women in this context experience heightened exposure to violence and intensified social stigma, which erodes the agency required to initiate recovery [21-24].

Finally, multivariable analysis demonstrated that readiness for change was socially patterned. Consistent with evidence linking early onset of substance use to more severe dependence, an earlier age at initiation was associated with poorer readiness for change in the present study [25-27]. Conversely, increasing age and current marriage were independently associated with higher “Taking Steps,” suggesting that life-course stability and relational roles may function as motivational anchors for behavioural change [28,29]. Finally, the association between daily injecting and reduced capacity for sustained recovery further underscores how high-frequency use patterns constrain the transition to action-oriented stages [30]. Together, these findings reinforce that readiness among FIDUs is not merely a cognitive state but a dynamic condition shaped by life-course positioning, relational stability, and cumulative exposure to injecting environments.

Limitations

The cross-sectional design precludes causal inference and limits assessment of changes over time. The study was conducted at a single centre, and participants were recruited through facility-based sampling, which may limit the generalizability of the findings to the broader population of FIDUs. In addition, the study was conducted in a context with a high proportion of female sex workers, and therefore, the findings may not be fully generalizable to all FIDU populations. Finally, reliance on self-reported data may introduce recall and social desirability biases.

## Conclusions

This study demonstrates that most FIDUs occupy a behavioural profile characterized by low recognition, moderate ambivalence, and fragile early action. Engagement in “taking steps” is shaped not only by individual insight but also by substance-use intensity, perceived inadequacy of services, comorbid health conditions, and socio-demographic constraints. Readiness for change in this population, therefore, reflects constrained agency within structurally vulnerable lives rather than indifference to harm.

In response, the findings support a shift from uniform, behaviour-focused interventions toward motivation-centred, stage-matched care. Routine assessment of readiness for change should be embedded within harm-reduction settings, accompanied by brief recognition-oriented counselling. Given the prominence of withdrawal fear and rapid relapse after short-lived attempts, programmes must prioritize structured withdrawal preparation, early clinical support, and continuity beyond crisis-driven care. Peer-supported, stage-based follow-up is essential to sustain fragile recovery efforts and prevent disengagement.

## Appendices

Appendix A

Questionnaire

Clinical examination and anthropometric assessment:

Height cm

Weight kg

Blood pressure mm/hg

BMI kg/m²

I. Socio-Demographic

1. What is your age?

_______ years

2. What is your marital status?

a. Never married

b. Married

c. Divorced/Separated

d. Widowed

3. What is your religion?

a. Christian

b. Hindu

c. Muslim

d. Other (Please specify): __________

4. Which tribe do you belong to?

a. Paite

b. Zou

c. Tangkhul

d. Mizo

e. Anal

f. Hmar

g. Other (Please specify): __________

5. What is your highest level of education?

a. Illiterate

b. No formal education

c. Primary school

d. Middle school

e. Secondary school

f. Higher secondary

g. Graduate and above

6. What is your current employment status?

a. Employed

b. Unemployed

7. What is your occupation?

_______ (Please specify)

8. What is your monthly income?

_______ (Please specify the amount)

9. What is your current living arrangement?

a. In a joint family

b. In a nuclear family (husband and kids)

c. Alone (at home)

d. With friends/co-workers at their home

e. Cohabitating with a partner

f. With co-workers at the workplace

g. Homeless

h. Any other (please specify): _______________

II. Drug Use and Consumption Patterns

1. Age at first use of injecting drug: _______________ (in years)

2. Reasons for initiating drug use:

a. Peer pressure

b. Introduced by drug-using husband/boyfriend

c. To overcome feelings of depression

d. To cope with separation from a romantic partner

e. Family issues or conflicts (e.g., to cope with family stress, parent-child conflict, domestic violence, others)

f. Curious about the effect of drugs

g. For fun

h. Somebody tricked me

i. Easy access to drugs as a peddler

j. Any other (please specify): _______________

10. Do you use substances only in injectable form?

a. Yes, I use only injectable substances.

b. No, I use substances in other forms as well.

11. What is the primary/most commonly injected substance you use?

a. Heroin (No.4)/Brown sugar

b. Buprenorphine (Tidigestic)

c. Pheniramine (Avil)

d. Benzodiazepine

e. Promethazine

f. Amphetamine-type stimulants

g. Any other (please specify): _______________

3. Do you inject drugs daily?

1) If Yes → How many times per day?

a. Once a day

b. Twice a day

c. Three times a day

d. Four times a day

e. More than four times a day

2) If No → How many times per week do you inject?

a. Once a week

b. 2-3 times a week

c. ≥4 times a week

12. What is your preferred route of injection?

a. Intravenous (IV)

b. Subcutaneous (SC)

c. Intramuscular (IM)

13. Monthly expenditure on drugs:

_______ (Please specify the amount)

III. Morbidity Profile

1. Injecting-Related Complications (last 3 months)

Have you ever experienced any of the following complications related to injecting drugs?

a. Abscesses/skin infection

b. Blocked veins

c. Overdose

d. Any other complications (please specify): _______________

2. Acute Health Problems (in the last 15 days)

Have you experienced acute health problems in the past 15 days? (Multiple responses)

3. Fever

4. Cold/Flu

5. Diarrhea

6. Headache

7. Body aches

8. Respiratory issues

9. Jaundice

10. Others (please specify_______________)

3. Chronic Health Problems/Comorbidities

Do you have any diagnosed chronic health problems? (Multiple responses)

1. Diabetes

2. Hypertension

3. Chronic respiratory diseases (e.g., asthma, COPD, Tuberculosis)

4. Chronic liver disease

5. Chronic kidney disease

6. Cardiovascular diseases (e.g., heart disease)

7. Mental health conditions (e.g., depression, anxiety)

8. Neurological conditions (e.g., Peripheral Neuropathy, autonomic hyperactivity)

9. Others (please specify_______________)

4. Reproductive Health Symptoms (last 3 months)

Have you suffered from any of the following? (Multiple responses)

a. Foul-smelling vaginal discharge

b. Genital ulcer/sore/itching

c. Lower abdominal excessive pain during or in between periods

d. Swelling in groin area

e. Burning while urinating

f. Genital warts

5. Serological status (within the last 1 year)

a. Hepatitis C: Positive/Negative/Status unknown

b. Hepatitis B: Positive/Negative/Status unknown

c. HIV: Positive/Negative/Status unknown

Appendix B

Interview Guide 1: Group 1 (High Recognition + High Taking Steps + Low Ambivalence)

Target Group: Female injecting drug users who fully acknowledge their drug use as a problem, are actively trying to change, and are certain about quitting (action or maintenance stage).

Goal: 10-15-minute interview, capturing motivations and barriers for sustained change.

Section 1: Recognition of the Problem

(Purpose: Confirm acknowledgment; informed by Miller & Rollnick, 1991, motivational interviewing)

1. What problems does injecting drugs cause you?

Probes:

• How does it affect your health (e.g., infections, tiredness)?

• What about relationships with family, partners, or friends?

• Does it make money or daily tasks harder?

• When did you realize these were issues?

2. What do people around you say about your drug use?

Probes:

• What do family, partners, or friends say?

• What about staff at the Drop-in Centre?

• Do their words motivate you to keep changing? How?

• Has anyone’s support been key to your efforts?

Section 2: Motivation

(Purpose: Understand drive for change; informed by Prochaska et al., 1992, action stage)

3. Why do you want to quit injecting drugs?

Probes:

• Is it for your health, family, future, or something else?

• What’s the strongest reason pushing you forward?

• Has a specific event or moment made you more determined?

• What keeps you certain about quitting?

4. What feels good about working to stop?

Probes:

• Do you feel stronger, healthier, or more hopeful?

• What’s the best part of this change so far?

• How do others (e.g., family, staff) react to your progress?

• Does anything make you even more committed?

Section 3: Taking Steps

(Purpose: Explore actions; informed by DiClemente et al., 2004, processes of change)

5. What are you doing to stop injecting drugs?

Probes:

• Are you using services like counseling, methadone, or needle exchange?

• Have you cut back, changed routines, or avoided certain places/people?

• What’s worked best for you?

• What tried but didn’t work, and why?

6. What helps you keep going with these changes?

Probes:

• Do people (e.g., family, friends, staff) support you? How?

• Does this centre or other services help? In what way?

• Are personal goals or beliefs driving you?

• What’s made the biggest difference?

Section 4: Barriers

(Purpose: Identify obstacles; informed by Rhodes et al., 2006, and Sharma et al., 2016, structural barriers)

7. What makes it hard to keep changing or get help?

Probes:

• Are there practical issues like money, transport, or time?

• Do you face stigma or judgment from others?

• Are police or legal issues a problem?

• How do these affect your progress?

8. As a woman, what’s the toughest part of getting support?

Probes:

• Do childcare, partners, or family duties get in the way?

• Are you afraid of being judged (e.g., by clinics, community)?

• Have you faced violence or threats tied to drug use?

• What would make support easier for women like you?

Closing Questions

1. Anything else you want to share about your journey?

2. Any questions for me about this study?

(Closing Statement)

Appendix C

Interview Guide 2: Group 2 (Low Recognition + Low Taking Steps + High Ambivalence)

Target Group: Female injecting drug users who don’t see their drug use as a major problem, aren’t trying to change, and feel conflicted about quitting (pre-contemplation or contemplation stage).

Goal: 10-15 minute interview, capturing motivations and barriers for early stages.

Section 1: Recognition of the Problem

(Purpose: Assess denial or minimization; informed by Miller & Rollnick, 1991, motivational interviewing)

1. Does injecting drugs cause any problems for you?

Probes:

• What about health (e.g., feeling sick, injuries)?

• Does it affect family, partners, or friends?

• Are money or daily tasks ever an issue?

• If it feels okay, why do you think that is?

2. What do people around you say about your drug use?

Probes:

• Do family, partners, or friends comment? What do they say?

• What about staff at the Drop-in Centre?

• Do their words make you think twice? Why or why not?

• Do you agree with them, or see it differently?

Section 2: Motivation

(Purpose: Explore ambivalence; informed by Prochaska et al., 1992, and Guise et al., 2017, contemplation stage)

3. Do you ever think about quitting or cutting back?

Probes:

• What makes you think about it (e.g., health, family, incidents)?

• What makes you hesitate or want to keep using?

• Are there moments you lean toward changing? Why?

• What pulls you back to staying the same?

4. How do you feel about the idea of changing?

Probes:

• Are you torn, scared, or unsure? Why?

• What’s the hardest part about considering change?

• Is there anything that makes changing seem possible?

• What would need to happen to make you think more about it?

Section 3: Taking Steps

(Purpose: Check for minimal actions; informed by DiClemente et al., 2004, early processes)

5. Have you ever tried to cut back or stop?

Probes:

• If yes, what did you try (e.g., using less, avoiding people)?

• What happened-did it last, or why did you stop trying?

• If no, what’s kept you from trying?

• How do you feel about the idea of trying?

6. What might help you consider changing?

Probes:

• Would people (e.g., family, friends) make a difference? How?

• Could this centre or other services help? In what way?

• Are there things in your life that need to change first?

• What would make starting easier?

Section 4: Barriers

(Purpose: Identify obstacles; informed by Rhodes et al., 2006, and Sharma et al., 2016, structural barriers)

7. What stops you from seeking help for drug use?

Probes:

• Are practical things like money or transport an issue?

• Do you worry about stigma or judgment?

• Are police or legal problems a concern?

• What makes help feel out of reach?

8. As a woman, what’s the toughest part of getting support?

Probes:

• Do childcare, partners, or family duties play a role?

• Are you scared of being judged (e.g., by clinics, community)?

• Have you faced violence or threats tied to drug use?

• What would make support better for women like you?

Closing Question

1. Anything else you want to share about your life?

2. Any questions for me about this study?

(Closing Statement)
